# Regulatory effect of traditional Chinese medicine on gut microbiota in patients with atherosclerosis: A protocol for systematic review and meta-analysis

**DOI:** 10.1097/MD.0000000000023730

**Published:** 2020-12-11

**Authors:** Langlang Huang, Jianan Wang, Ri Xu, Yanwei Liu, Zhongyong Liu

**Affiliations:** a Jiangxi University of Traditional Chinese Medicine; b The Affiliated Hospital of Jiangxi University of Traditional Chinese Medicine, Nanchang, Jiangxi Province, PR China.

**Keywords:** atherosclerosis, gut microbiota, protocol, systematic review, traditional Chinese medicine

## Abstract

**Background:**

Atherosclerosis is the pathological basis of many cardiovascular and cerebrovascular diseases, and its pathogenesis is complex. Recent studies revealed a significant role of gut microbiota in the onset and development of atherosclerosis. Traditional Chinese medicine has rich clinical experience and unique advantages in the treatment of atherosclerosis. A large number of studies have proved that traditional Chinese medicine has the functions of reducing blood lipid, regulating gut microbiota, and resisting inflammation. The aim of this systematic review is to observe the randomized controlled trial of traditional Chinese medicine in treating gut microbiota, so as to evaluate the effectiveness and safety of traditional Chinese medicine in treating atherosclerosis patients.

**Methods:**

The English database (PubMed, Web of Science, Embase, the Cochrane Library) and Chinese database (China National Knowledge Infrastructure, the Chongqing VIP Chinese Science, and Technology Periodic Database, Wanfang Database, and China Biomedical Literature Database) will be searched up to October 2020. We will also manually search the Chinese clinical trial register, conference papers, and unpublished studies or references. Randomized control trials of traditional Chinese medicine treatment of atherosclerosis were collected comprehensively, and 2 researchers will independently screen literature, data extraction, and evaluation the quality of literature methodology. The primary outcomes are lipid metabolism and gut microbiota and their metabolites. The secondary outcomes are the change of inflammatory markers. Meta-analysis was performed by RevMan 5.3.5 software. The Grades of Recommendation, Assessment, Development, and Evaluation will be used to evaluate the outcome quality of evidence.

**Results:**

This study will comprehensively review the existing evidence of traditional Chinese medicine in treating atherosclerosis from the perspective of gut microbiota.

**Conclusion:**

This study will provide information on the effectiveness and safety of traditional Chinese medicine in treating atherosclerosis from the perspective of gut microbiota.

**Unique INPLASY number:**

INPLASY2020110056.

## 1 Introduction

Atherosclerosis (AS) is a chronic inflammatory disease related to lipid accumulation and changes in a blood vessel wall components, which occurs in large and medium arteries. It is the pathological basis of many cardiovascular and cerebrovascular diseases such as ischemic heart disease, cerebral infarction, and cerebral hemorrhage. With the development of the social economy and the aggravation of population aging, its incidence rate increases, which causes higher mortality and social pressure in the world.^[1]^ According to statistics, about 17 to 18 million people die of atherosclerotic cardiovascular disease every year in the world, accounting for 31% of all deaths; In China, the number of cardiovascular disease patients reached 300 million.^[2,3]^ The pathogenesis of AS is unclear, and the risk factors include smoking, drinking, hypertension, hyperlipidemia, and obesity, among which abnormal lipid metabolism is the most critical risk factor.^[4–6]^ This disease's pathogenesis is complex, involving lipid infiltration, endothelial injury, platelet hyperfunction, inflammatory reaction, oxidative stress, and so on.^[7]^ However, neither theory can fully explain the formation mechanism of AS alone.

There are more than 3,500 kinds of gut microbiota (GM) in normal human intestines, with a total of about 100 trillion. GM's stable composition and function play an essential role in maintaining the intestinal tract's normal physiological part and immune defense.^[8]^ With the deepening of related research, more and more evidence shows that GM imbalance can lead to the formation of AS by participating in the regulation of cholesterol metabolism, oxidative stress, and inflammation^[9–11]^; Moreover, GM can also participate in the metabolism of nutrients such as protein, dietary fiber, and choline in vivo, and produces metabolites such as trimethylamine N-oxide (TMAO), short-chain fatty acids), and bile acids (BAs) to regulate the immunity and metabolism of host, thus affecting the occurrence and development of AS.^[12,13]^

Traditional Chinese medicine (TCM), as the crystallization of Chinese conventional wisdom, has multi-component, multi-target, and multi-channel characteristics and plays a unique role in clinical prevention and treatment of diseases. Many modern pharmacological studies have proved that TCM has the functions of protecting vascular endothelial cells, resisting platelet aggregation, reducing blood lipid, resisting inflammation, resisting oxidative stress, and regulating GM.^[14–18]^ After oral administration of TCM, its practical components are mainly absorbed in the intestinal tract, and stay in the intestinal tract for a long time, which is beneficial to regulating GM diversity and metabolism. Moreover, TCM can also play an anti-AS role by regulating the metabolites of GM, such as reducing the production of TMAO and increasing the ratio of lactic acid bacteria bifidobacteria to promote BA metabolism and slow down the AS process.^[19]^

In recent years, although many reviews have summarized the effects of TCM on GM and AS and related clinical trials and basic research have been carried out,^[20,21]^ there is no systematic review of the effects of TCM on AS from the perspective of GM regulation. The purpose of this study is to systematically review randomized controlled trials (RCTs), so as to evaluate the evidence of regulating effect and curative effect of TCM on GM of patients with AS.

## 2 Methods

### 2.1 Protocol and registration

The protocol has been registered on the INPLASY website, and the registration number is INPLASY2020110056 (URL https://inplasy.com/inplasy-2020-11-0056/). This report will be performed by the Preferred Reporting Items for Systematic Review and Meta-Analysis Protocols.^[22]^

### 2.2 Literature search

We will search the following databases from their inception onwards to the October 2020: PubMed, Web of Science, Embase, the Cochrane Library, China National Knowledge Infrastructure, the Chongqing VIP Chinese Science and Technology Periodical Database, Wanfang Database, and China Biomedical Literature Database. We will also manually search the Chinese Clinical Trial Register, conference papers, and unpublished studies or references. The search strategy for PubMed is listed in Table 1, a similar method will be applied to the Chinese database.

**Table 1:** PubMed search strategy.

Number	Search terms
#1	Atherosclerosis[MeSH]
#2	Atheroscleroses[title/abstract]
#3	Atherogenesis[title/abstract]
#4	#1 OR #2 OR #3
#5	Gastrointestinal Microbiome[MeSH]
#6	Gut Microbiota[title/abstract]
#7	Microbiome, Gastrointestinal[title/abstract]
#8	Gut Microbiome[title/abstract]
#9	Gut Microbiomes[title/abstract]
#10	Microbiome, Gut[title/abstract]
#11	Gut Microflora [title/abstract]
#12	Microflora, Gut[title/abstract]
#13	Gut Microbiotas[title/abstract]
#14	Microbiota, Gut[title/abstract]
#15	Gastrointestinal Flora[title/abstract]
#16	Flora, Gastrointestinal[title/abstract]
#17	Gut Flora[title/abstract]
#18	Flora, Gut[title/abstract]
#19	Gastrointestinal Microbiota[title/abstract]
#20	Gastrointestinal Microbiotas[title/abstract]
#21	Microbiota, Gastrointestinal[title/abstract]
#22	Gastrointestinal Microbial Community[title/abstract]
#23	Gastrointestinal Microbial Communities[title/abstract]
#24	Microbial Community, Gastrointestinal[title/abstract]
#25	Gastrointestinal Microflora[title/abstract]
#26	Microflora, Gastrointestinal[title/abstract]
#27	Gastric Microbiome[title/abstract]
#28	Gastric Microbiomes[title/abstract]
#29	Microbiome, Gastric[title/abstract]
#30	Intestinal Microbiome[title/abstract]
#31	Intestinal Microbiomes[title/abstract]
#32	Microbiome, Intestinal[title/abstract]
#33	Intestinal Microbiota[title/abstract]
#34	Intestinal Microbiotas[title/abstract]
#35	Microbiota, Intestinal[title/abstract]
#36	Intestinal Microflora[title/abstract]
#37	Microflora, Intestinal[title/abstract]
#38	Intestinal Flora[title/abstract]
#39	Flora, Intestinal[title/abstract]
#40	Enteric Bacteria[title/abstract]
#41	Bacteria, Enteric[title/abstract]
#42	#5 OR #6 OR #7 OR #8 OR #9 OR #10 OR #11 OR #12 OR #13 OR #14 OR #15 OR #16 OR #17 OR #18 OR #19 OR #20 OR #21 OR #22 OR #23 OR #24 OR #25 OR #26 OR #27 OR #28 OR #29 OR #30 OR #31 OR #32 OR #33 OR #34 OR #35 OR #36 OR #37 OR #38 OR #39 OR #40 OR #41
#43	Medicine, Chinese Traditional[MeSH]
#44	Traditional Chinese Medicine[title/abstract]
#45	Chung I Hsueh[title/abstract]
#46	Hsueh, Chung I[title/abstract]
#47	Traditional Medicine, Chinese[title/abstract]
#48	Zhong Yi Xue[title/abstract]
#49	Chinese Traditional Medicine[title/abstract]
#50	Chinese Medicine, Traditional[title/abstract]
#51	Traditional Tongue Diagnosis[title/abstract]
#52	Tongue Diagnoses, Traditional[title/abstract]
#53	Tongue Diagnosis, Traditional[title/abstract]
#54	Traditional Tongue Diagnoses[title/abstract]
#55	Traditional Tongue Assessment[title/abstract]
#56	Tongue Assessment, Traditional[title/abstract]
#57	Traditional Tongue Assessments[title/abstract]
#58	#43 OR #44 OR #45 OR #46 OR #47 OR #48 OR #49 OR #50 OR #51 OR #52 OR #53 OR #54 OR #55 OR #56 OR #57
#59	randomized controlled trial[Publication Type]
#60	randomized[Title/Abstract]
#61	placebo[Title/Abstract]
#62	#59 OR #60 OR #61
#63	#4 AND #42 AND #58 AND #62

### 2.3 Inclusion criteria

According to the Participants’ principle, Intervention, Comparison, and Outcome, the following standards are formulated.

#### 2.3.1 Type of studies

RCTs on TCM intervention in treating AS patients will be included in this review.

#### 2.3.2 Type of participants

Patients who meet the diagnostic criteria for AS will be included.

#### 2.3.3 Types of interventions

The treatment group was treated with TCM or TCM combined with western medicine.

#### 2.3.4 Type of comparators

The control group will receive western medicine treatment or without intervention and was not treated with TCM.

#### 2.3.5 Types of outcome measures

The lipid metabolism outcome indicators (TG, TC, HDL-C, LDL-C, HDL, LDL, ApoA1, ApoB) and GM and its metabolites (GM structure and diversity, TMAO, short-chain fatty acids, BA) are the primary outcome indicators of this study. This study's secondary outcome include carotid intima-media thickness, atherosclerotic plaque area, inflammatory factor level (such as TNF-α, CRP, IL-6), safety index, and incidence of adverse events.

### 2.4 Exclusion criteria

(1) TCM combined with other non-drug adjuvant therapies (such as tai chi, acupuncture, and moxibustion);
(2) Documents with similar original data and repeated publication;
(3) Documents for which accurate data cannot be obtained;
(4) Case report, conference papers, and summaries;
(5) No RCT

### 2.5 Studies selection

**Figure 1:**
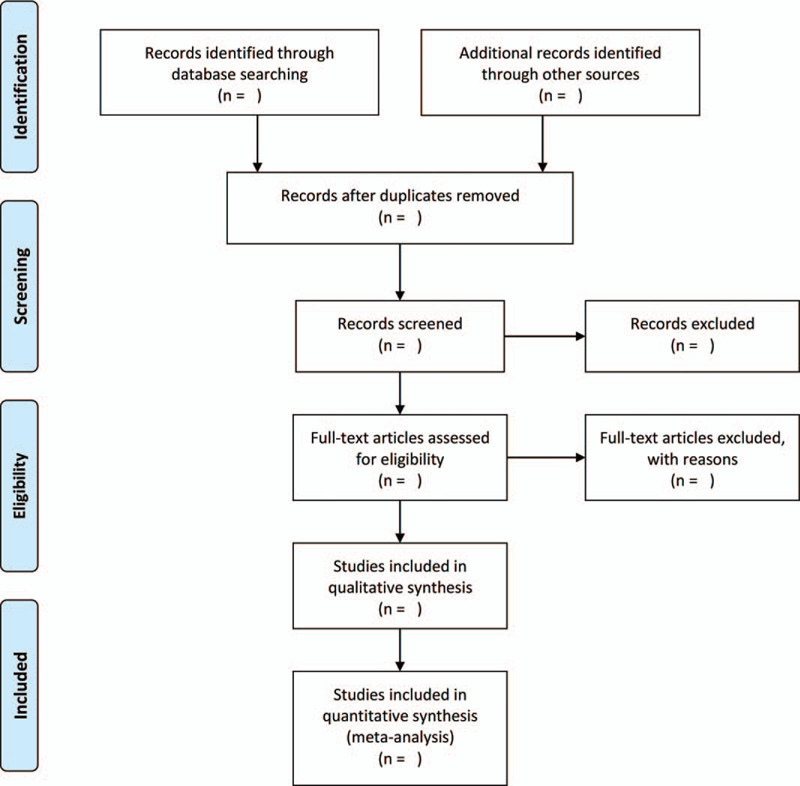
Flow diagram of literature retrieval.

Two researchers in the research group independently searched out the literature. First, identical pieces of literature were screened and excluded by Endnote X9 document management software. The summary of the title exclusion system was read, and finally, the full text was read, which met the inclusion criteria. In case of disagreement, the third author discussed and decided together. The selection process will be shown according to the Preferred Reporting Items for Systematic Review and Meta-Analysis flow chart in Figure 1.

### 2.6 Data extraction and management

After the literature was included, 2 other researchers independently completed the data extraction, including the author's name, year of publication, title, country, average age, gender, study design, the total number of cases, participants, intervention measures, comparison, outcome, and any other relevant information.

### 2.7 Quality assessment of the studies

According to the bias risk evaluation standard of randomized controlled trials provided by Cochrane Handbook,^[23]^ the literature quality evaluation is carried out, which includes the following 6 aspects: random sequence generation, allocation concealment, blinding of participants, caregivers, outcome assessors, incomplete outcome data, selective outcome reporting, and other bias. According to the specific scoring rules, the 2 researchers evaluated 3 types: “low risk,” “high risk,” and “uncertain risk.” If there is any difference in quality evaluation, discuss it with the third author.

### 2.8 Measures of treatment effect

According to the different variables, the dichotomous variables are expressed as risk ratio and 95% confidence intervals. Continuous variables are defined as weighted mean difference or standardized mean difference and 95% confidence intervals.

### 2.9 Management with missing data

If there are insufficient or lost data, we will first contact the original author by email or telephone. If the lost data cannot be obtained, we will discard the unusable data and only analyze the available data.

### 2.10 Assessment of heterogeneity

The heterogeneity test adopts *P* ≤ .10 and *I*^*2*^ ≥ 50% as the significance judgment criteria, and when *P* > .10 and *I*^*2*^ < 50%, the fixed effects models will be used for data analysis. If *P *≤ .10 and *I*^*2*^ ≥ 50%, the random effects model will be chosen.

### 2.11 Data synthesis

RevMan5.3.5 software will be used for this meta-analysis. According to the heterogeneity level included in the study, the fixed-effect model or the random effect model was selected. We will conduct a meta-analysis of at least 3 qualified standards. Otherwise, if only 1 or 2 studies met the inclusion criteria, meta-analysis will not be undertaken, but the descriptive analysis will be adopted. If more than 10 articles are included, the inverted funnel diagram is used to analyze publication bias.

### 2.12 Sensitivity analysis

We consider sensitivity analysis for methodological quality and test the results’ robustness by excluding the risk of low quality and high bias.

### 2.13 Subgroup analysis

If heterogeneity is observed in the study, we will use subgroup analysis for research.

### 2.14 Summary of evidence

The evidence quality of each result is evaluated by the method of Recommendation, Assessment, Development, and Evaluation.^[24]^ The evaluation will be divided into 4 qualities: “very low,” “low,” “medium,” or “high.”

### 2.15 Ethical approval and dissemination

Ethical approval is not necessary for this study because there is no individual data will be used. The research results will be published in a peer-reviewed journal and conference presentations.

## 3 Discussion

AS belongs to the category of “Mai Bi” in TCM. The theory of TCM holds that the interior and exterior of the heart and intestine, so it is possible to treat heart diseases by regulating GM. TCM has less toxic and side effects in the prevention and treatment of AS. With the development of sequencing technology and metagenome research, many studies have confirmed that TCM can regulate GM and its metabolites and resist AS. However, there is no systematic evaluation of RCTs on the role of TCM in AS treatment from the perspective of GM. Therefore, we hope that this study can provide the latest evidence for the effectiveness and safety of TCM in treating AS and regulating of GM and its metabolites and being used to guide clinical practice.

## Author contributions

**Conceptualization:** Langlang Huang, Zhongyong Liu.

**Data curation:** Langlang Huang, Jianan Wang, Ri Xu.

**Formal analysis:** Jianan Wang, Ri Xu, Yanwei Liu.

**Investigation:** Langlang Huang, Zhongyong Liu.

**Methodology:** Langlang Huang, Jianan Wang, Ri Xu, Yanwei Liu.

**Software:** Jianan Wang, Yanwei Liu.

**Supervision:** Langlang Huang, Zhongyong Liu.

**Writing – original draft:** Langlang Huang, Jianan Wang, Yanwei Liu, Zhongyong Liu.

**Writing – review & editing:** Langlang Huang, Jianan Wang, Ri Xu.
